# Genome-wide identification of grain filling genes regulated by the OsSMF1 transcription factor in rice

**DOI:** 10.1186/s12284-017-0155-4

**Published:** 2017-04-26

**Authors:** Joung Sug Kim, Songhwa Chae, Kyong Mi Jun, Yoon-Mok Pahk, Tae-Ho Lee, Pil Joong Chung, Yeon-Ki Kim, Baek Hie Nahm

**Affiliations:** 1 Division of Bioscience and Bioinformatics, Myongji University, Yongin, Kyonggido 449-728 Republic of Korea; 2 Genomics Genetics Institute, GreenGene BioTech Inc., Yongin, Kyonggido 449-728 Republic of Korea; 3 Department of Agricultural Biotechnology, National Institute of Agricultural Science, 370 Nongsaengmyeong-ro, Wansan-gu, Jeonju, North Jeolla Province 54874 Republic of Korea; 4 Crop Biotechnology Institute, GreenBio Science and Technology, Seoul National University, Pyeongchang, 25354 Republic of Korea

**Keywords:** OsSMF1, DNA-binding motif, Transcription factor, Grain filling genes

## Abstract

**Background:**

Spatial- and temporal-specific expression patterns are primarily regulated at the transcriptional level by gene promoters. Therefore, it is important to identify the binding motifs of transcription factors to better understand the networks associated with embryogenesis.

**Results:**

Here, we used a protein-binding microarray (PBM) to identify the binding motifs of OsSMF1, which is a basic leucine zipper transcription **f**actor involved in the regulation of rice **s**eed **m**aturation. *OsSMF1* (previously called *RISBZ1 or OsbZIP58*) is known to interact with GCN4 motifs (TGA(G/C)TCA) to regulate seed storage protein synthesis, and it functions as a key regulator of starch synthesis. Quadruple 9-mer-based PBM analysis and electrophoretic mobility shift assay revealed that OsSMF1 bound to the GCN4 (TGA(G/C)TCA), ACGT (CCACGT(C/G)), and ATGA (GGATGAC) motifs with three different affinities. We predicted 44 putative OsSMF1 target genes using data obtained from both the PBM and RiceArrayNet. Among these putative target genes, 18, 21, and 13 genes contained GCN4, ACGT, and ATGA motifs within their 1-kb promoter regions, respectively. Among them, six genes encoding major grain filling proteins and transcription factors were chosen to confirm the activation of their expression in vivo. OsSMF1 was shown to bind directly to the promoters of Os03g0168500 (GCN4 motif), patatin-like gene (GCN4 motif), α-globulin (ACGT motif), rice prolamin box-binding factor (RPBF) (ATGA motif), and ONAC024 (GCN4 and ACGT motifs) and to regulate their expression.

**Conclusions:**

The results of this study suggest that *OsSMF1* is one of the key transcription factors that functions in a wide range of seed developmental processes with different specific binding affinities for the three DNA-binding motifs.

**Electronic supplementary material:**

The online version of this article (doi:10.1186/s12284-017-0155-4) contains supplementary material, which is available to authorized users.

## Background

Transcription is known to be regulated by the binding of transcription factors to their cognate motifs in the promoter regions of genes. In monocotyledons, several *cis*-elements, such as the prolamin box (TGTAAAG), GCN4 motif (TGA(G/C)TCA), and AACA motif (AACAAAA), are highly conserved in the promoters of seed storage protein (SSP)-encoding genes and play central roles in controlling endosperm-specific gene expression during seed maturation (Takaiwa et al. 1996; Wu et al. 2000) Maize opaque-2 (O2) is an endosperm-specific transcription factor belonging to the bZIP family that has been shown to bind to the ACGT motif of the maize 22-kDa *zein* promoter and activate transcription (Schmidt et al. 1992). In addition, a barley bZIP transcriptional activator (BLZ1) binds to the GCN4 motif, which is putatively involved in regulating gene expression in the endosperm (Vicente-Carbajosa et al. 1998). Further, rice endosperm bZIP, RISBZ2 (also called REB), and opaque-2 heterodimerizing protein 1 (OHP1) specifically bind to the GCCACGT(A/C)AG sequence in the *α-globulin* (*α-Glb*) gene promoter and the TCCACGTAGA sequence in the 22-kDa *zein* promoter, respectively (Nakase et al. 1997; Pysh et al. 1993). Other transcription factors are also involved in regulating the expression of starch synthesis genes. For example, a MYC-like protein (OsBP-5) and OsEBP-89, a member of the ethylene-responsive element-binding protein (EREBP) family, act synergistically as a heterodimer to regulate transcription of the rice *Wx* gene (Zhu et al. 2003). Additionally, *rice starch regulator 1* (*RSR1*), which is an EREBP-type transcription factor, negatively regulates starch biosynthesis, and an *RSR1-*deficient mutant has been shown to exhibit the enhanced expression of starch synthesis genes in seeds (Fu and Xue 2010).

*OsSMF1* (previously called *RISBZ1*) is a basic leucine zipper transcription **f**actor that is involved in the regulation of rice **s**eed **m**aturation. It belongs to the maize O2-like protein group and is known to interact with the GCN4 motif (TGA(G/C)TCA) to regulate SSPs (Onodera et al. 2001). A previous study has shown that *OsSMF1* (alternatively named *OsbZIP58*) is also involved in seed development, including the regulation of starch biosynthesis genes (Wang et al. 2013). It specifically binds to the ACGT elements in the promoters of both *granule-bound starch synthase* (*GBSS*) and *starch branching enzyme* (*SBE1*) (Wang et al. 2013). *OsSMF1* gene expression is restricted to seeds, where it precedes the expression of storage protein-encoding genes (Onodera et al. 2001). Studies have demonstrated that *OsSMF1* is involved in the regulation of starch synthesis, in addition to that of SSP synthesis, in the endosperm (Onodera et al. 2001; Wang et al. 2013). Here, we aimed to identify target genes for further functional analysis of OsSMF1.

For the large-scale identification of transcription factor target sites, several laboratory techniques, such as chromatin immunoprecipitation (ChIP) and DNA adenine methyltransferase identification (DamID), have been devised (Orian 2006; Pokholok et al. 2002; Ren et al. 2000; van Steensel et al. 2001; Wyrick et al. 2001). Recently, chip-based protein-binding microarrays (PBMs) have been developed, allowing for the identification of protein-DNA interactions in vitro. In our previous study, we constructed a quadruple 9-mer protein-binding microarray (Q9-PBM), in which 131,072 quadruple probes were designed to cover all possible combinations of 9-mers of the reverse complementary sequences (Kim et al. 2009). The specificity of Q9-PBM was confirmed using well-known DNA-binding sequences, including Cbf1 and CBF1/DREB1B, and it was also used to elucidate the unidentified *cis*-acting element of the *ONAC024* rice transcription factor (Kim et al. 2009). The Q9-PBM has mainly been used to assess the interactions of transcription factors with short synthetic DNA sequences and to evaluate their DNA sequence specificities. Here, we used the Q9-PBM to determine the binding motifs of OsSMF1. In a previous study, the RiceArrayNet (RAN) was developed from collective data obtained from 60 K rice microarrays (Lee et al. 2009), and it has been widely used (Hamada et al. 2011; Lorenz et al. 2011; Movahedi et al. 2011). We have constructed a new version of the RAN that provides co-expression information on genes, including correlation coefficients, calculated using accumulated data obtained from 300 K rice microarrays (http://bioinfo.mju.ac.kr/arraynet/rice300k_2011/query/). To predict the co-expressed genes, we also examined the relationships among genes that are putatively regulated by *OsSMF1* by RAN analysis.

Q9-PBM analysis revealed that OsSMF1 recognized three binding motifs, namely the GCN4 [TGA(G/C)TCA], ACGT [CCACGT(G/C)], and ATGA (GGATGAC) motifs, with different affinities. We detected 85 genes that were positively regulated by OsSMF1 using the RAN analysis under the tested conditions, with a minimum correlation value of 0.55 and a depth of 1. Among these putative target genes, 18 (21.2%), 21 (24.7%), and 13 (15.3%) contained GCN4, ACGT, and ATGA motifs within their 1-kb promoter regions, respectively. In addition to confirming the known OsSMF1 target genes, we predicted 35 potential target genes that have not been previously described in immature seeds. Using qRT-PCR and the protoplast transactivation assay, we found that in vivo, OsSMF1 activated *Os03g0168500* and *patatin-like protein*, which contain the GCN4 motif; *ONAC024* and *ONAC026*, which contain either the GCN4 or ACGT motifs; and *prolamin box-binding factor* (*RPBF*) and *CCCH-type zinc finger protein* (*OsGZF1*), which contain the ATGA motif. The results suggest that *OsSMF1* has specific binding affinities for the three motifs and that it functions in a wide variety of seed developmental processes.

## Results

### Identification of the multiple binding motifs of OsSMF1 by PBM

**Fig. 1:**
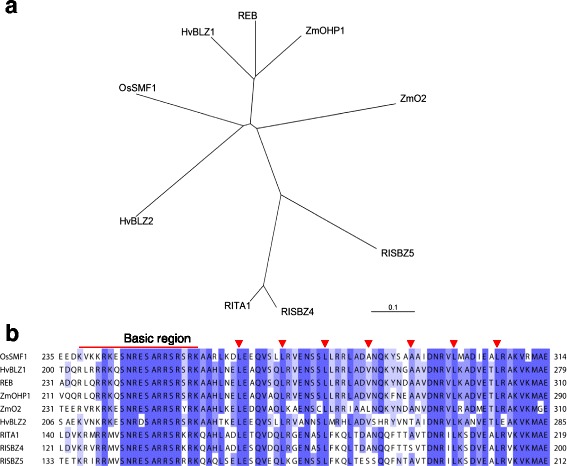
Phylogenetic and amino acid comparisons of bZIP transcription factors. **a** Phylogenetic tree of bZIP transcription factors from *Oryza sativa* (OsSMF1: LOC_Os07g08420, REB: LOC_Os03g58250, RITA1: LOC_Os02g16680, RISBZ4: LOC_Os02g07840, and RISBZ5: LOC_Os06g45140), *Zea mays* (ZmO2: CAA34614 and ZmOHP1: EF144174), and *Hordeum vulgare* (HvBLZ1: CAA56374 and HvBLZ2: CAA71795). The phylogenetic tree was constructed using Clustal Omega (http://www.ebi.ac.uk/Tools/msa/clustalo/) with full-length sequences, including transit peptides, and it was visualized using TreeView program. **b** Alignment of the deduced amino acid sequence of the conserved bZIP domain of OsSMF1 with those of related plant bZIP proteins. The basic region and leucine repeats are indicated by the *bar* and *arrows*, respectively

*OsSMF1* is a transcription factor previously known as *RISBZ1* or *OsbZIP58.* In this study, *OsSMF1* was mainly expressed in the endosperm at 11 and 21 DAF during seed maturation (Additional file 1: Figure S1) (Onodera et al. 2001; Wang et al. 2013). A previous classification of bZIP proteins from green plants resulted in the identification of five rice bZIP transcription factors, including OsSMF1, belonging to the same group (Correa et al. 2008). In this study, sequence analyses were performed using ClustalW, and the results revealed that OsSMF1 possessed a high degree of similarity of 52.7% with the REM (RISBZ2) protein, whereas it shared approximately 31% sequence identity with the RITA-1 (RISBZ3), RISBZ4, and RISBZ5 proteins (Fig. 1a). Additionally, it possessed approximately 51.3 and 49.6% amino acid sequence identities with maize OHP1 and barley BLZ1, respectively, which contained conserved sequences within the leucine zipper regions with homologies of 73.7–76.3% (Fig. 1b). These endosperm-specific transcription factors with similarities to OsSMF1 are known to bind to either the GCN4 or ACGT motif.

We first attempted to identify the binding motifs of the OsSMF1 protein using a Q9-PBM. This transcription factor, as well as similar variants, bind to multiple binding motifs (Godoy et al. 2011). To this end, the full-length *OsSMF1* cDNA was fused at the N-terminus to the *DsRed* fluorescent protein. Purified recombinant OsSMF1-DsRed protein expressed in *E. coli* was hybridized to the Q9-PBM. Then, the consensus binding motifs were determined based on signal strength (Jung et al. 2012; Kim et al. 2009).

**Fig. 2:**
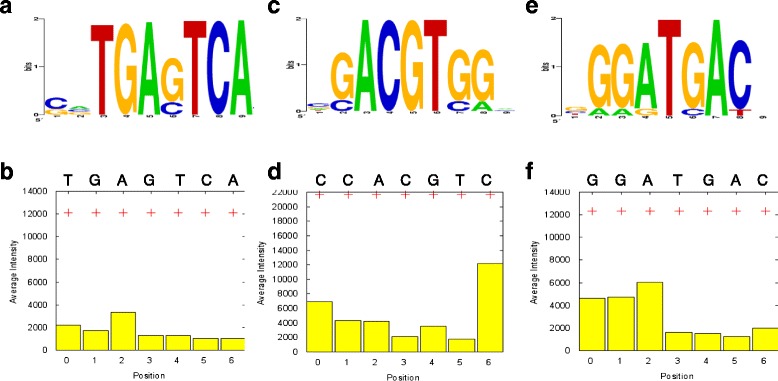
Identification of binding motifs of recombinant OsSMF1 from *E. coli* by Q9-PBM. **a, c, e** The consensus binding sequences according to the binding intensities observed on the Q9 protein-binding microarray. The overall consensus binding motif was obtained by multiple alignments of the significant binding sequences with ClustalW, and it was visualized with SeqLogo program. **b, d, f** The effects of mutations on the binding intensities of the consensus binding motif. The average binding intensities (+) of probes containing TGAGTCA (**b**), CCACGTC (**d**), and GGATGAC (**f**) were plotted. The *yellow boxes* represent the average binding intensities of probes substituted at each position with other bases

The signal distribution curve of the OsSMF1 PBM was characterized by a deep leftward slope, followed by a right tail (Additional file 2: Figure S2); the shape of the curve was attributed to specific interactions between the protein and features on the microarray. For motif extractions, 1,286 total signals in the steep left region with high intensities were clustered and identified using SEQLOGO (Fig. 2a, c and e). We found significant binding motifs for OsSMF1, including ACGT (CCACGTCA), with a high intensity of 13,715, GCN4 (TGAGTCA) with a moderate intensity of 7,639, and ATGA (GGATGAC) with an intensity of 6,463 (Fig. 2a, c and e). In addition, we found that OsSMF1 bound to a new motif termed the ATGA motif, which can be considered a novel OsSMF1 binding motif. To determine the flanking sequence of the ACGT core sequence that is essential for DNA binding by OsSMF1, we analyzed the relative signal intensities of single nucleotide substitution variants of the putative binding sequences (Fig. 2b). In agreement with the Q9-PBM results, individual substitutions at all positions of the CCACGTC sequence resulted in significantly reduced binding signal intensities. Although C had a stronger binding affinity than G at the seventh nucleotide in the ACGT motif, G also had a relatively strong affinity for OsSMF1. Thus, we found a significantly high affinity of OsSMF1 for the ACGT motif, CCACGT(G/C), which may be considered an OsSMF1-specific binding motif (Fig. 2).

**Fig. 3:**
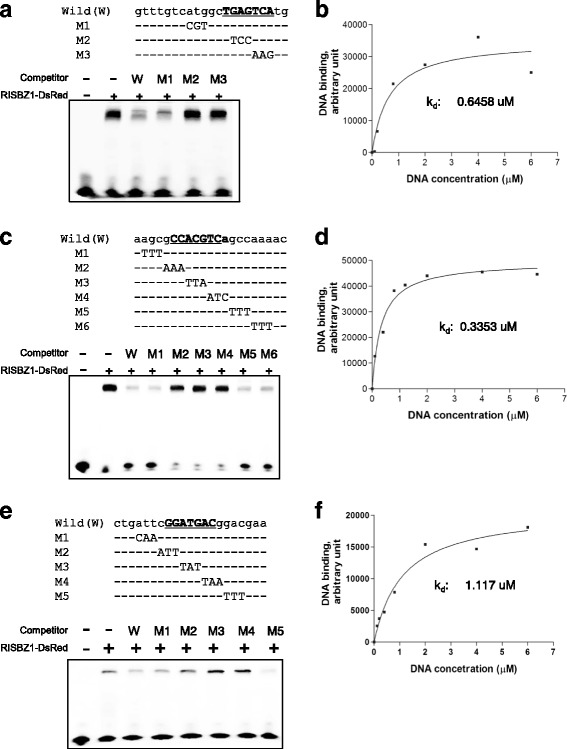
Identification of OsSMF1 binding motifs in vitro. **a, c, e** EMSA-based competition analysis of OsSMF1. Sequences of oligonucleotides used as probes and competitors are depicted. Wild type, 21-bp sequences containing the GCN4 (**a**), ACGT (**c**), and ATGA motifs (**e**); and M1 to M6, 21-bp sequences with three nucleotide mutations. The GCN4, ACGT, and ATGA motifs are underlined. The OsSMF1-DsRed fusion protein was used for EMSA with the 21-bp sequences. Competitors were added in 100-fold molar excess. Lane 1, no protein; Lane 2, no competitor; and lanes 3 to 9, with competitors. **b, d, f** Accurate determination of the dissociation constant *K*_d_ by gel shift assays using SYBR Gold. The DNA-binding band intensities determined using Gel-Pro Analyzer program were plotted against the various DNA substrate concentrations. The measured *K*_d_ values were 0.6458 μM for the binding of OsSMF1 to the GCN4 motif (**b**), 0.3353 μM for ACGT (**d**), and 1.117 μM for ATGA (**f**)

To further analyze the multiple binding motifs of OsSMF1 with different intensities, we assayed their binding specificities to recombinant OsSMF1 by electrophoretic mobility shift assay (EMSA) using biotinylated double-stranded oligonucleotide probes corresponding to each motif. The binding of OsSMF1 to the 21-bp fragments of the GCN4, ACGT, and ATGA motifs was detected as lagging bands (Fig. 3). As shown in Fig. 3a, c and e, three bases of the 21-bp motifs were sequentially mutagenized, and these mutants were used as competitors (100-fold increased molarities) in the EMSAs. The results revealed that the introduction of mutagenized nucleotides into any part of the core motifs as competitors had little or no effect on the binding of native fragments, whereas mutations flanking the core motifs led to the loss of binding. Taken together, these results suggested that OsSMF1 specifically bound to the GCN4, ACGT, and ATGA motifs with high affinity (Fig. 3a, c and e). For the determination of binding affinities, dissociation constants (*K*_*d*_) were estimated by gel shift assay using SYBR Gold. DNA-binding band intensity was measured at various DNA substrate concentrations (0, 0.1, 0.2, 0.4, 0.8, 1.2, 2, 4, and 6 μM) in the presence of OsSMF1 (0.2 μg/μl). The *K*_*d*_ values for the binding of OsSMF1 to the GCN4, ACGT, and ATGA motifs were 0.6458 μM, 0.3353 μM, and 1.117 μM, respectively (Fig. 3b, d and f). The binding affinities of OsSMF1 for the three DNA-binding motifs, as indicated by the *K*_*d*_ values, showed the same trends as those revealed by the Q9-PBM, indicating that OsSMF1 was able to bind to the GCN4, AGCT, and ATGA motifs independently with different affinities.

### Gene expression network of OsSMF1 based on microarray expression data

OsSMF1 is considered an important transcription factor involved in the regulation of SSPs and starch synthesis. To further elucidate the regulatory mechanisms of OsSMF1 in the cereal endosperm, we predicted its target genes. In a previous paper, the RAN was developed using accumulated data from 60 K rice microarrays (Lee et al. 2009), and the new version of the RAN has been used to elucidate gene functions, as it provides co-expression information for genes, including correlation coefficients, obtained from 174 300 K microarrays of the rice genome (*Oryza sativa*) (http://bioinfo.mju.ac.kr/arraynet/rice300k_2011/) (Hamada et al. 2011; Lorenz et al. 2011; Movahedi et al. 2011). We identified 85 genes that were positively correlated with *OsSMF1* in rice using the RAN under the tested conditions, with a minimum correlation value of 0.55 and a depth of 1 (Additional file 3: Table S2, Additional file 4: Figure S3). Among them, 21 (24.7%), 18 (21.2%), and 13 (15.3%) putative target genes contained ACGT, GCN4, and ATGA motifs in their 1-kb promoter regions, respectively (Table 1).

**Table 1:** Candidate OsSMF1 target genes, the binding motifs and RAN analysis and microarray expression data

IRGSP Gene	r-value	Motif						Description
		GCN4		ACGT		ATGA		
		motif position	motif sequence	motif position	motif sequence	motif position	motif sequence	
Os01g0762500	0.63	889	tgagtca					GluA-1
Os10g0400200	0.56	897	tgagtca					GluA-2
Os01g0898500	0.64	773	tgagtca					Patatin-like protein 2 (PAT)
Os06g0133000	0.55	185	tgagtca					Granule-bound starch synthase I, chloroplast precursor (GBSS)
Os03g0168500	0.63	496	tgagtca					Endosperm-specific gene 44 (OsEnS44)
Os03g0712800	0.63	200	tgagtca					Glutamine synthetase root isozyme 2
Os01g0393400	0.62	572	tgagtca					MDR-like ABC transporter
Os03g0799600	0.59	702	tgagtca					ES43-like protein
Os04g0437000	0.58	512	tgagtca					No apical meristem (NAM) domain-containing protein (ONAC79)
Os02g0459400	0.55	272	tgagtca					Conserved hypothetical protein
Os03g0179700	0.54	932	tgagtca					Protein of unknown function DUF567 family protein
Os03g0427300	0.66	898	tgagtca	381	ccacgtc			GluA-3
Os05g0415400	0.55	850	tgagtca	352	ccacgtg			No apical meristem (NAM) domain-containing protein (ONAC024)
Os04g0528000	0.66	716	tgagtca	366	ccacgtg			Protein of unknown function DUF789 family protein
Os02g0268100	0.59	832	tgagtca			171	ggatgac	GluB-5
Os02g0268300	0.57	818	tgagtca			185	ggatgac	GluB-4
Os09g0520400	0.63	715	tgagtca			744	ggatgac	OsbZIP76
Os02g0249500	0.61	105	tgagtca			802	ggatgac	Hypothetical protein
Os05g0499100	0.55			436	ccacgtc			26-kDa globulin (alpha-globulin)
Os07g0182000	1.00			841	ccacgtc			OsSMF1
Os11g0582400	0.56			100	ccacgtc			Endosperm-specific protein (OsEnS146)
Os01g0393100	0.60			257	ccacgtc			No apical meristem (NAM) domain-containing protein (*ONAC026*)
Os05g0569400	0.63			650	ccacgtg			Galactose mutarotase-like domain-containing protein.
Os01g0133400	0.61			381	ccacgtg			Hexose transporter (fragment)
Os02g0694100	0.59			534	ccacgtc			Cyclin-like F-box domain-containing protein
Os03g0598700	0.59			32	ccacgtc			Zn-finger, RING domain-containing protein
Os05g0404700	0.59			342	ccacgtc			Methyl-CpG-binding protein (MBD1)
Os11g0701100	0.59			832	ccacgtg			Xylanase inhibitor protein 2
Os01g0585100	0.58			966	ccacgtc			Conserved hypothetical protein
Os06g0141400	0.57			10	ccacgtc			Early nodulin
Os05g0557600	0.57			226	ccacgtc			Ubiquitin-conjugating enzyme domain-containing protein
Os04g0508500	0.56			397	ccacgtc			Snapdragon myb protein 305 homolog
Os05g0491400	0.56			196	ccacgtc			LRR protein
Os01g0517800	0.56			862	ccacgtg			PLATZ transcription factor
Os04g0629300	0.55			263	ccacgtc			SNF2-related domain-containing protein
Os10g0138600	0.69			240	ccacgtc	158	ggatgac	Cyclin-like F-box domain-containing protein
Os12g0151000	0.69			426	ccacgtg	233	ggatgac	Purple acid phosphatase
Os02g0252400	0.66					737	ggatgac	Rice prolamin box-binding factor (RPBF)
Os07g0668600	0.65					360	ggatgac	Zinc finger, CCCH-type domain-containing protein (OsGZF1)
Os04g0677400	0.66					975	ggatgac	Conserved hypothetical protein
Os07g0457400	0.62					764	ggatgac	Conserved hypothetical protein
Os03g0766200	0.61					770	ggatgac	Bifunctional inhibitor/plant lipid transfer protein/seed storage domain-containing protein
Os07g0485100	0.60					589	ggatgac	Nitrilase/cyanide hydratase and apolipoprotein N-acyltransferase domain-containing protein
Os07g0603600	0.56					237	ggatgac	Alpha/beta hydrolase family protein

These predicted target genes were functionally categorized into 4 groups according to the Gene Ontology analysis (http://geneontology.org/) (Table 2). Among the 44 predicted genes, 6 were categorized under the term “nutrient reservoir activity (GO:0045735)”. Among these 6 genes, five glutelin genes (GluA-1, GluA-2, GluA-3, GluB-4, and GluB-5) contained the GCN4 motif with or without the other motifs, and one globulin gene (α-glb) contained the ACGT motif. Previous reports (Kawakatsu et al. 2008; Yamamoto et al. 2006) have indicated that OsSMF1 initiates *trans*-activation from the promoters of the *GluA-1*, *GluA-2*, and *GluA-3* genes following recognition of the GCN4 motif. *GBSS*, a known target gene of OsSMF1, was also identified in our analysis (Wang et al. 2013). Our results consistently demonstrate the involvement of OsSMF1 not only in starch synthesis but also in the regulation of SSPs in developing seeds. Five and three additional genes were functionally categorized under the terms “defense response (GO:0006952)” and “negative regulation of RNA metabolic process” (GO:0051253), respectively (Table 2). Further, 10 transcription factors, including OsSMF1, were assigned to "regulation of nitrogen compound metabolic process (GO:0051171).” Among these transcription factors, two, including OsGZF1 and rice prolamin box-binding factor (RPBF), were co-expressed with OsSMF1 (Chen et al. 2014; Yamamoto et al. 2006). These in vitro results suggest that the OsSMF1 gene may be involved in the regulation of a wide range of target genes involved in seed development during embryogenesis.

**Table 2:** Gene Ontology classification of putative OsSMF1 target genes

Accession	Name	Ontology	IRGSP Gene	motif			Description
				GCN4	ACGT	ATGA	
GO:0045735	nutrient reservoir activity	molecular_function	Os01g0762500	O			GluA-1
			Os10g0400200	O			GluA-2
			Os03g0427300	O	O		GluA-3
			Os02g0268300	O		O	GluB-4
			Os02g0268100	O		O	GluB-5
			Os05g0499100		O		26-kDa globulin (alpha-globulin)
GO:0051171	regulation of nitrogen compound metabolic process	biological_process	Os07g0182000		O		OsSMF1
			Os01g0393100		O		No apical meristem (NAM) domain-containing protein (*ONAC026*)
			Os05g0404700		O		Methyl-CpG-binding protein (MBD1)
			Os04g0629300		O		SNF2-related domain-containing protein.
			Os05g0415400	O	O		No apical meristem (NAM) domain-containing protein (ONAC024)
			Os03g0799600	O			ES43-like protein
			Os04g0437000	O			No apical meristem (NAM) domain-containing protein (ONAC79).
			Os09g0520400	O		O	OsbZIP76
			Os02g0252400			O	Rice prolamin box-binding factor (RPBF)
			Os07g0668600			O	CCCH-type Zinc finger protein (OsGZF1)
GO:0006952	defense response	biological_process	Os11g0582400		O		Endosperm-specific protein (OsEnS146)
			Os11g0701100		O		Xylanase inhibitor protein 2
			Os03g0179700	O			Protein of unknown function DUF567 family protein
			Os02g0268100	O		O	GluB-5
			Os02g0268300	O		O	GluB-4
GO:0051253	negative regulation of RNA metabolic process	biological_process	Os05g0404700		O		Methyl-CpG-binding protein (MBD1)
			Os03g0799600	O			ES43-like protein
			Os04 g0629300		O		SNF2-related domain-containing protein

### Expression of target genes enhanced by overexpression of *OsSMF1*

To assess whether the putative target genes described above are biologically significant in vivo, a vector was constructed in which *OsSMF1* was expressed under control of the promoter of *Wsi* (a member of the group 3 *Lea* family), which is predominantly active in the whole grain, including the endosperm, embryo, and aleurone layer, during seed development (Yi et al. 2011). Microarray analysis further showed high *Wsi* mRNA expression in the callus (Additional file 1: Figure S1). The bialaphos resistance (*bar*) gene was used as a selectable marker to identify transgenic calli. Four independent transgenic plants were obtained using the *Agrobacterium*-mediated transformation method (Sohn et al. 2006). Calli were generated from T1 seeds, and the expression of *OsSMF1* and its target genes was measured by RT-PCR and qRT-PCR.

**Fig. 4:**
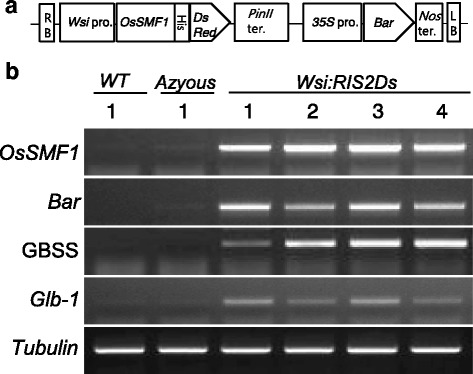
Production of DsRed-fused OsSMF1-transformed rice. **a** Schematic representation of the expression vector *Wsi:SMF1* used for rice transformation. *Wsi* pro, rice responsive to ABA protein promoter; *PinII* ter, potato proteinase inhibitor II terminator; *35S* pro, cauliflower mosaic virus 35S promoter; *Bar*, phosphinothricin acetyltransferase; and *Nos* ter, nopaline synthesis terminator. **b** The expression of OsSMF1 and its target genes was determined by RT-PCR analysis. Specifically, the expression of granule-bound starch synthase (GBSS) *and* 26-kDa globulin (*α-Glb*) were analyzed. Total RNA was extracted from calli. *Tubulin* expression is shown as a constitutive control

**Fig. 5:**
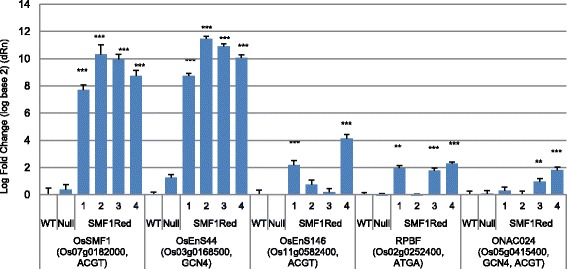
Expression of four genes in wild-type and OsSMF1-transformed rice, as determined by real-time PCR. The expression levels of genes in W*si:SMF1Red* calli after normalization to *Tubulin* was compared with those in wild-type calli. The results shown are the mean ± SD; *n* = 3 (^***^ *P* < 0.001; **P < 0.01; and *P < 0.05 compared with wild type)

The overexpression of *OsSMF1* was confirmed by RT-PCR and qRT-PCR, which demonstrated that expression of this gene was upregulated by 200- to 1000-fold in calli derived from Os*SMF1*-transformed plants compared with that in calli derived from wild-type and nullizygous plants (Figs. 4 and 5). We also confirmed that the expression of *α-Glb* and *GBSS*, which are known target genes of *OsSMF1* but were not expressed in the calli, was increased in *OsSMF1*-transformed calli compared with that in nullizygous and wild-type calli, as shown by RT-PCR (Fig. 4). These results revealed that OsSMF1 activated expression of the *α-Glb* and *GBSS* genes in vivo.

The expression of *Os03g0168500* (endosperm-specific gene 44, *OsEnS44*) was markedly upregulated in four independent calli derived from OsSMF1-transformed plants compared with that in nullizygous and wild-type calli (Fig. 5). The promoter region of *OsEnS44* contains GCN4 motifs in its 1-kb promoter region. *Os11g0582400* (endosperm-specific protein 146, *OsEnS146*) expression was also significantly increased in two independent OsSMF1-transformed calli (Fig. 5), and its promoter region contains the ACGT motif. Thus, our results indicated that *OsEnS44* and *OsEnS146* acted as novel OsSMF1 target genes in vivo. Two transcription factors identified as putative OsSMF1 target genes, *RPBF* and *ONAC024,* were selected for validation of their expression in the *OsSMF1*-transformed calli by qPCR. The promoter region of *ONAC024* contains both the GCN4 and ACGT motifs, and the ATGA motif has been detected in the RPBF promoter. *RPBF* expression was increased by up to 4-fold in three independent *OsSMF1*-transformed calli compared with that in wild-type calli (Fig. 5). Further, *ONAC024* expression was upregulated by approximately 2-fold in two independent *OsSMF1*-transformed calli (Fig. 5). These results also indicated that *RPBF* and *ONAC024* could be considered target genes of OsSMF1. Previous studies have suggested that RPBF and OsSMF1 (RISBZ1) synergistically activate seed-specific genes during grain filling (Kawakatsu et al. 2009; Yamamoto et al. 2006).

### Binding analysis of promoter regions targeted by OsSMF1

To investigate whether OsSMF1 activates expression of the putative target genes listed in Table 2, we performed a transient activation assay in rice protoplasts using 13 putative genes: *α-Glb*, *patatin-like protein, prolamin10.2, OsEnS44, OsEnS146, ONAC024, ONAC026, OsSMF1, aldose 1-epimerase family protein, methyl-CpG-binding protein (MBD1), PLATZ, RPBF, and OsGZF1.* Their promoters were fused to *fLUC* (*promoter:fLUC*) as a reporter. Among the 13 putative genes, six genes were upregulated and one gene, OsGZF1, was downregulated by OsSMF1.

**Fig. 6:**
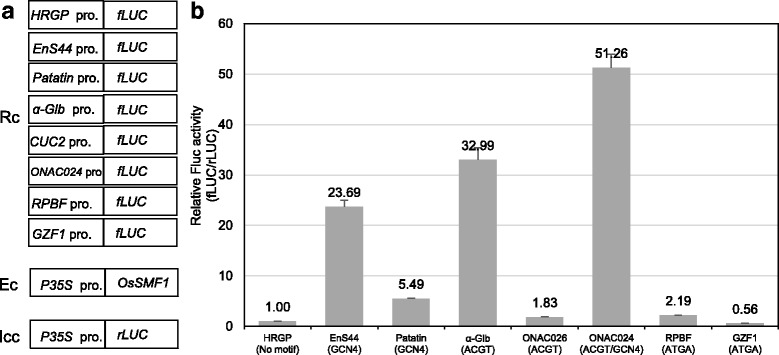
Transcriptional activity analysis of OsSMF1 in rice protoplast using a dual luciferase assay system. **a** Construction of reporter (Rc), effector (Ec), and internal control (Icc) vectors. **b** P35S:SMF1 and P35S:rLUC constructs were co-transfected with *TRX:fLUC*, *HRGP:fLUC* or *α-Glb:fLUC* into rice protoplasts. rLUC was used as an internal control. The data are presented as the mean ± SD (*n* = 3)

The expression of *OsEnS44* and *OsEnS146*, which contain the GCN4 and ACGT motifs in its promoter region, respectively, was dramatically increased in OsSMF1-transformed rice. *RPBF* and *ONAC024* transcription factors, the promoters of which contain the ATGA and GCN4/ACGT motifs, respectively, were highly expressed in OsSMF1-transformed calli compared with wild-type calli. We also selected *patatin-like protein*, the promoter of which contains the GCN4 motif (Table 1), as a storage protein. The other selected genes, including *patatin-like protein*, showed similar expression patterns in both transgenic and wild-type calli (data not shown). The *α-Glb* promoter was used as a positive control. The *Os12g0621600* (**h**ydroxyproline-**r**ich **g**lyco**p**rotein, *HRGP*) promoter, which was determined to be correlated with *OsSMF1* in RAN analysis (correlation value of 0.6) but does not contain any of the three binding motifs in its promoter, was selected as a negative control (*HRGP:fLUC*). In these assays, the effector gene, *OsSMF1*, was expressed under control of the P35S promoter and co-transfected together with the reporter construct into the protoplast. Analysis of LUC activity demonstrated that the co-transfection of *EnS44:fLUC*, *α-Glb:fLUC*, and *ONAC024:fLUC* with *OsSMF1* resulted in induction of the expression of the luciferase reporter gene by 23.7-, 33-, and 51.3-fold, respectively, compared with co-transfection of *HRGP:fLUC* (Fig. 6). The activity of fLUC by the promoters of the *Patatin-like protein*, *ONAC026*, and *RPBF* were increased up to 5.5-, 1.8-, and 2.2-fold in the presence of OsSMF1, but the promoter of *OsGZF1* was downregulated by this transcription factor (Fig. 6). These results indicated that OsSMF1 regulated the expression of *OsEnS44* and *patatin-like* genes by binding to the GCN4 motifs in their promoters. The expression of *RPBF* and *ONAC026* was also directly regulated by the binding of OsSMF1 to the ATGA and ACGT motifs in the promoter, respectively. The expression of *ONAC024,* which contained both the GCN4 and ACGT motifs in its promoter, was greatly increased by OsSMF1 (Fig. 6).

## Discussion

Determination of the DNA-binding specificities of transcription factors is important for the understanding of transcriptional regulatory codes. Rice seed development is directly related to crop yield and is finely controlled by complex regulatory networks. Gene co-expression network analysis has shown that transcription factors are involved in the complex regulation of rice seed development (Xue et al. 2012). However, many of the primary target genes of these transcription factors as part of regulatory networks remain to be elucidated. We aimed to identify target genes of the seed-specific transcription factor OsSMF1, which is known to be involved in the regulation of rice SSP gene expression (Kawakatsu et al. 2009; Yamamoto et al. 2006) and starch synthesis (Onodera et al. 2001; Wang et al. 2013). Q9-PBM analysis, which is a powerful and rapid method for the identification of putative functional *cis*-regulatory elements, allows for the accurate quantification of the binding affinities of some transcription factor binding motifs using all possible 9-mer combinations of probes (Kim et al. 2009). We used this technology to characterize the binding specificities of OsSMF1 and identified three DNA-binding motifs for this transcription factor, including the GCN4 (TGA(G/C)TCA), ACGT (CCACGT(C/G)), and ATGA (GGATGAC) motifs (Figs. 2 and 3). It has recently become apparent that a transcription factor may interact with more multiple DNA elements. For example, *Arabidopsis* MYC2 binds with high affinity to the G-box (CACGTG), T/G (AACGTG), and G-like (CATGTG) motifs (Godoy et al. 2011). Another rice bZIP protein, REM, binds to both the ACGT and GCN4 motifs (Nakase et al. 1997). OsSMF1 and REM have a close phylogenetic relationship (52.9%). However, OsSMF1 expression is restricted to the mature seed, whereas REM is expressed in all tissues. Furthermore, the expression of *OsSMF1* at 11 DAP was determined to be 6-fold higher than that of *REM* in this study (Additional file 1: Figure S1). These results suggest that *OsSMF1* plays important roles in a wide range of biosynthetic processes during seed maturation. In addition to the known GCN4 binding motif, OsSMF1 also recognizes the ATGA and ACGT motifs. The ACGT motif has been previously shown to be a target of OsSMF1-regulated gene expression, but the essential flanking sequence of this motif has not yet been identified (Wang et al. 2013). Q9-PBM analysis and EMSA performed in this study revealed that CCACGT(C/G) is an essential sequence that is bound by OsSMF1 (Figs. 2 and 3). We found that OsSMF1 bound to TGAGTCA (GCN4 motif), CCACGTC (ACGT motif), and GGATGAC (ATGA motif) with intensities of 6,463, 13,715, and 7,639, respectively. The *K*_*d*_ value for the binding of OsSMF1 to the ACGT motif was approximately two- and four-fold higher than those for the GCN4 and ATGA motifs, respectively (Fig. 3), demonstrating that OsSMF1 plays prominent roles during seed development by binding to these three motifs in vitro.

First, we selected genes containing the GCN4, ACGT, and ATGA motifs in their 1-kb promoter regions from the RAP2 rice database. Second, the RAN analysis, which was based on the expression pattern correlation, was capable of narrowing down the putative target genes of *OsSMF1* to a subset of 85 genes (Additional file 2: Figure S2). To validate these predicted genes, we searched the GCN4 motif for known OsSMF1 binding sequences by combined microarray analysis. Among the six putative genes, four genes (*GluA-1*, *GluA-2, GluA-3*, and *α-Glb*) have been previously identified as OsSMF1 target genes by transient assay using rice callus protoplasts (Yamamoto et al. 2006). Among the remaining targets that were detected, two globulin genes (*GluB-4* and *GluB-5*) were characterized as SSPs. A previous study demonstrated that OsSMF1 causes reduced activation of the *α-Glb* promoter compared with that of the *GluA-1* and *GluA-3* promoters (Yamamoto et al. 2006). However, this group assessed the *α-Glb* region from −340 bp to +73 bp, which does not include the ACGT motif positioned at −436 bp relative to the ATG start codon, in transient assays using rice callus protoplasts. Although patatin-like protein was not classified under the Gene Ontology term “nutrient reservoir activity (GO:0045735)”, it contained the GCN4 motif and was determined to be one of the direct target genes of OsSMF1 (Table 1, Fig. 6). Patatin represents approximately 40% of the soluble protein in potato tubers, but it has not yet been studied in rice.

Among the 44 total putative target genes, 9 transcription factors (27.5%), including *OsSMF1*, were observed, which were assigned to “regulation of nitrogen compound metabolic process (GO:0051171, Table 2). *OsSMF1* is specifically expressed in the aleurone and subaleurone layers of the developing endosperm (Onodera et al. 2001), and the expression of the target genes of this transcriptional activator may be restricted to these subcellular locations. Among the 9 transcription factors, 6 genes, which were preferentially expressed at 11 and 21 DAP when *OsSMF1* was also highly expressed (Additional file 5: Figure S4), were selected and four transcription factors were identified as target genes through in vivo protoplast analysis. The promoters of *OsSMF1*, *ONAC026*, and *MBD1* contained the ACGT motif, and the *OsGZF1* and *RPBF* genes incorporated the ATGA motif in their promoters. The *ONAC024* transcription factor, which contained both the GCN4 and ACGT motifs on its promoter, showed significantly increased expression by OsSMF1. *ZmaNAC36*, which was cloned from maize by homologous cloning using rice *ONAC026*, is co-expressed with many starch synthesis genes. Thus, *ONAC026* might be co-expressed with starch biosynthetic genes in the rice endosperm. A previous study showed that *OsSMF1* (alternatively named *OsbZIP58*) null mutants displayed abnormal seed morphology with altered starch accumulation and decreased amounts of total starch, particularly amylase, with binding to the promoters of six starch-synthesizing genes (Wang et al. 2013). We suggest that *ONAC024* and *ONAC026* might be additional transcription factors that regulate the synthesis of starch in the presence of OsSMF1 during seed development. In addition, both the ACGT and GCN4 motifs in the *ONAC024* promoter provides a high-affinity binding site for *OsSMF1* compared to the ACGT motif in the *ONAC026* promoter. *OsGZF1* is known to repress the activation of OsSMF1, in addition to promoting the down regulation of *GluB-1* expression (Chen et al. 2014). Protoplast assay revealed that luciferase activity of the *GZF1:Fluc* vector was decreased by 0.5-fold compared with that of the negative control (Fig. 6). Expression of the *RPBF* transcription factor was increased in the *OsSMF1*-transformed calli compared with that in wild-type plants (Fig. 5), and OsSMF1 bound to the promoter of *RPBF* in vivo, as determined by protoplast assay (Fig. 6). Additionally, RPBF and OsSMF1 have been reported to synergistically activate transcription from the promoters of rice SSPs (Kawakatsu et al. 2009; Yamamoto et al. 2006). Knock-out transgenic rice, in which the accumulation of OsSMF1 (called RISBZ1) and RPBF was reduced, showed a significant reduction in SSPs (Kawakatsu et al. 2009). These results suggest that OsSMF1 directly regulates the expression of *OsGZF1* and *RPBF* by binding to the ATGA motif in their promoters to regulate SSPs. However, neither *OsSMF1* nor *MBD1* was expressed by OsSMF1 in the protoplast assay (data not shown). The protoplast in vivo analysis revealed that the binding of OsSMF1 alone to the binding motif was not sufficient to self–activate the OsSMF1 transcription factor (data not shown). This result eliminated the possibility that OsSMF1 autoregulates itself as observed with GUS reporter genes under the control of the OsSMF1 promoter in rice protoplast (Onodera et al. 2001).

The majority of the newly identified target genes have unknown functions in seed development. We showed that two unknown target genes were highly expressed in *OsSMF1*-transformed calli compared with wild-type calli. Among them, *OsEnS44* was identified as an OsSMF1 target gene. Interestingly, we determined that OsSMF1 bound to the promoter of *OsEnS44* by protoplast transactivation assay (Fig. 6). OsEnS44 contains a thioredoxin domain, which acts in redox regulation throughout the life cycle of the seed (Wong et al. 2003). The activation of OsEnS44 by OsSMF1 may be one transcription factor cascade or may act as a transcription factor for the activation of downstream genes that regulate the biosynthesis of the major seed components. Therefore, these results provide novel information regarding the regulatory roles of OsSMF1 in seed maturation. It can be concluded that OsSMF1 is one of the key transcription factors involved in grain filling that functions by regulating the expression of a wide variety of genes during seed maturation.

## Conclusions

Overall, the Q9-PBM results showed that OsSMF1 bound to TGAGTCA (GCN4 motif), CCACGTC (ACGT motif), and GGATGAC (ATGA motif) with different affinities. First, the transcription factor OsSMF1 regulated the expression of RPBF and OsGZF1, which contained the ATGA motif on their promoters. This result suggested that OsSMF1 regulated SSPs by binding the promoter of these genes. Second, OsSMF1 also regulated starch synthesis by binding to the promoters of *ONAC024 and ONAC026,* which contained either the ACGT or GCN4 motifs. Finally, we identified *OsEnS44* as an OsSMF1 target gene, which contained the GCN4 motif in its promoter region, but its function is unknown in seed development. Further studies on the regulatory mechanisms of OsSMF1, including phenotype observations in transgenic rice, will help advance the understanding of rice seed development.

## Methods

### Plasmid construction

*OsSMF1* was isolated from panicles of *Oryza sativa* cv. Ilmi by RT-PCR prior to the heading stage using the primers indicated in Additional file 6: Table S1. The full-length coding region of the gene was cloned into a pET-DsRed expression vector (Kim et al. 2012) to generate an *OsSMF1-DsRed* fusion gene, thereby producing the plasmid pET-SMFRed. This vector was used for the expression and purification of the OsSMF1 protein in *Escherichia coli*.

The *GFP* sequence of *Wsi18:GFP* (Yi et al. 2011) was replaced with a Gateway® cassette containing attR recombination sites flanking the *ccdB* gene and a chloramphenicol-resistance gene, thereby yielding the plasmid pSB-WsiGW. The *OsSMF1-DsRed* gene was then inserted into the pSB-WsiGW binary vector by a BP reaction following the manufacturer’s instructions (Invitrogen), generating the plasmid *Wsi:SMF1Red.* Finally, the constructs were introduced into *Agrobacterium tumefaciens* LBA4404 by triparental mating (Hiei et al. 1994).

### Protein expression and purification

The proteins were expressed in the *E. coli* strain BL21-CodonPlus (Stratagene). Cells were cultured overnight and inoculated into fresh liquid LB medium, grown at 37 °C to an OD_260_ of 0.6, and induced with 1 mM isopropyl β-D-1-thiogalactopyranoside (IPTG) at 25 °C for 5 h. Cell pellets were obtained by centrifugation at 4 °C for 5 min at 5,000 g, followed by washing with cold PBS buffer containing a protease inhibitor (Roche). Then, the cells were resuspended in 5 ml lysis buffer (50 mM NaH2PO4, 300 mM NaCl, and 10 mM imidazole (pH 8.0)), sonicated five times with 15 s bursts and 45 s of cooling on ice, and centrifuged at 14,000 g for 30 min at 4 °C. The resulting supernatants were incubated with 2 ml Ni-NTA agarose (Qiagen) for 30 min, loaded into empty columns, and washed twice with 5 ml lysis buffer containing 10 mM imidazole according to the manufacturer’s instructions. Purified proteins were eluted with 0.5 ml lysis buffer containing 240 mM imidazole. Five micrograms of purified proteins, as measured using the Bradford assay, were used in each experiment.

### Protein-binding microarray design

A microarray was designed as described previously and manufactured by Agilent Technology (Santa Clara, CA, USA) (Kim et al. 2009). This microarray (Q9-PBM) consisted of 232,145 quadrupled probes, including 131,072 probes for all possible 9-mers, 101,073 of which were replicated. Each 9-mer was concatenated four times, followed by a sequence complementary to a primer (5′-CGGAGTCACCTAGTGCAG-3′) and a 5-nt thymidine linker for attachment to the slide. Each microarray slide contained a total of 243,504 spots arranged in 267 columns and 912 rows. In addition to the quadruple probes, 1,474 random sequences from the yeast genome, 8,081 blank probes and 1,804 probes provided by the manufacturer were included.

### Protein-binding microarray experiments

Complementary DNA was synthesized and analyzed to verify successful synthesis according to previously described methods (Kim et al. 2009). A double-stranded microarray was washed with PBS–0.01% (v/v) Triton X-100 and blocked with PBS-2% (wt/v) BSA (Sigma) for 1 h. Then, it was washed with PBS–0.1% (v/v) Tween-20, PBS–0.01% (v/v) Triton X-100 and PBS for 1 min in each solution. Next, a protein binding mixture was prepared containing 200 nM TF in PBS-2% (wt/v) BSA, 51.3 ng/μl salmon tested DNA (Sigma) and 50 μM zinc acetate. The mixture was incubated with the microarray for stabilization of and hybridization with the probes at 25 °C for 1 h. Subsequently, the microarray was washed with PBS-50 μM zinc acetate-0.5% (v/v) Tween-20 for 10 min, PBS-50 μM zinc acetate-0.01% Triton X-100 for 2 min and PBS-50 μM zinc acetate for 2 min. Finally, fluorescence images were captured using a microarray scanner (Axon).

### Motif extraction

The consensus binding sequence was determined based on the fluorescence signal strength according to previously described methods (Jung et al. 2012; Kim et al. 2009). Two independent linear models (y = ax + b) were applied to the steep left and extended right tail regions of the rank-ordered fluorescence signal distribution curve for the bound protein using R statistical language. Spots that exhibited strong fluorescence and high enrichment were subjected to alignment. These groups of sequences were visualized with SEQLOGO ‘Visualize information content of patterns’ [http://www.bioconductor.org/packages/release/bioc/html/seqLogo.html], which yielded an intensity profile figure, sequence logos and related statistical data. P-values and position weight matrices were calculated using the Wilcoxon-Mann–Whitney test.

### Electrophoretic mobility shift assay (EMSA)

Biotin end-labeled and unlabeled oligonucleotides were annealed with each complimentary sequence (Additional file 6: Table S1). Five micrograms of OsSMF1 protein were incubated with 40 fmol biotin-labeled double-stranded oligonucleotides, 1 μg poly dI-dC, 1X binding buffer, 2.5% (v/v) glycerol and 0.05% (wt/v) NP-40 in a 20-μl reaction volume for 1 h at room temperature, according to the manufacturer’s instructions (Pierce). The reaction mixture was then analyzed by electrophoresis on a non-denaturing 6% acrylamide gel with 0.5X TBE buffer. Subsequently, the DNA-protein complexes in the gel were transferred to a positively charged nylon membrane by electrophoretic transfer in 0.5X TBE at 380 mA for 30 min, cross-linked at 120 mJ/cm^2^ using a UV light cross-linker, and detected using a Lightshift^TM^ Chemiluminescent EMSA Kit (Pierce).

### Analysis of DNA binding by EMSA

The reactions were conducted using various DNA substrate concentrations (0, 0.1, 0.2, 0.4, 0.8, 1.2, 2, 4, and 6 μM) and 0.2 μg/μl OsSMF1. Binding was performed in the presence of 10 mM Tris, 50 mM KCl, 1 mM DTT (pH 7.5), 50 ng/μl poly(dI–dC), 2.5% glycerol, 0.05% NP-40, and 5 mM MgCl_2_, and incubation for 1 h at room temperature. Following incubation, 2 μl of 10X EMSA loading dye was added to the reaction mixtures and loaded in an 8% polyacrylamide gel. The gel was stained with a SYBR Gold solution for 30 min and observed under UV light. DNA-binding band intensity was assessed using Gel-Pro Analyzer program, and the intensity value was used to calculate the *K*_*d*_ value using Prism software. The data were fit to the following equation:

$$ \mathrm{Y} = \mathrm{Bmax}\ *\ \mathrm{X}\ /\ \left({K}_d + \mathrm{X}\right) $$

where Bmax is the maximal binding, and *K*_*d*_ is the concentration of ligand required to reach half-maximal binding.

### *Agrobacterium*-mediated transformation of rice

The *Agrobacterium* strain LBA4404 harboring *Wsi:SMF1* was introduced into embryogenic rice calli (*Oryza sativa* L. Japonica cv. Ilmi) by *Agrobacterium*-mediated transformation (Sohn et al. 2006). Callus induction, co-cultivation with *A. tumefaciens*, and the selection of transformed calli were performed as previously described (Sohn et al. 2006). Selected calli were subcultured for 2 weeks on fresh 2 N6 medium with 250 mg/L cefotaxime, and then transferred to MSR medium containing 250 mg/L cefotaxime and 4 mg/L phosphinothricin and incubated for 4 weeks at 27 °C under continuous light for selection and regeneration. Regenerated shoots were transferred to MSO medium containing 4 mg/L phosphinothricin and incubated for 4 weeks for root induction. The plantlets were then transplanted to a Wagner pot (200 cm^2^) kept in a greenhouse for subsequent growth. *Wsi:SMF1* transgenic rice of the T_1_ generation were used in further analyses.

### RNA isolation and RT-PCR

Total RNA was extracted from leaves of transgenic and wild-type rice plants using TRI REAGENT® (Molecular Research Center, www.mrcgene.com) and purified with a Qiagen RNeasy Mini Kit (Qiagen, www.qiagen.com). cDNA templates were synthesized using RevertAid H minus M-MulV reverse transcriptase (Fermentas, www.fermentas.com). Semiquantitative RT-PCR was performed in a 20 μl reaction mixture under the following conditions: one cycle at 95 °C for 2 min and 25 to 35 cycles at 94 °C for 30 s, 60 °C for 30 s, and 72 °C for 30 s. Real-time quantitative RT-PCR analysis was performed using 2X Real-Time PCR Premix with EvaGreen (SolGent, www.solgent.com) according to the manufacturer’s protocol. Thermal cycling and fluorescence detection were performed using a Stratagene Mx3000P Real-Time PCR machine and Mx3000P software v2.02 (Stratagene, (http://www.genomics.agilent.com). Melting curve analysis (with an increase from 55 to 65 °C in increments of 0.1 °C s^−1^) was performed to ensure that only the desired PCR product was measured at a specific melting temperature. Real-time PCR was performed in triplicate for each cDNA sample. Following amplification, the PCR data were assessed using a comparative quantification (calibrator) analysis with Mx3000P software v2.02. (Stratagene). The rice *tubulin* gene (Os11g0247300) was used as an endogenous control. All primer pairs used are listed in Table S2.

### Transcriptional activity assay in rice protoplast

Transcriptional activity assay of OsSMF1 in rice protoplast was conducted using a dual luciferase reporter assay system (Promega, USA). To construct reporter plasmids, promoters of putative target genes of OsSMF1 were amplified from *Oryza sativa* cv. Ilmi genomic DNA using specific primers (Additional file 6: Table S1) and were then cloned into a pHBT vector (GenBank accession no. EF090408) between the firefly luciferase (fLUC) gene and nos terminator, respectively. For dual luciferase assay, the OsSMF1 and Renilla luciferase (rLUC) coding sequences were cloned into the pHBT vector between the *35S* promoter and *nos* terminator, respectively.

The resulting construct, P*35S:OsSMF1,* was co-transformed with the reporter plasmid into isolated rice protoplasts by polyethylene glycol (PEG)-mediated transformation (1 μg per transfection). *P35S:rLUC* (Renilla luciferase) was also added to each sample as an endogenous control (1 μg per transfection). Protoplast isolation and PEG-mediated transformation were performed as previously described (Jung et al. 2015). Luciferase activity was detected using an Infinite® 200 (Tecan, Switzerland). Measured fLUC activities were normalized to rLUC activities.

### Statistical analyses

All statistical analyses were performed using Sigma Plot v10 software.

## Additional files

Additional file 1: Figure S1. Transcript levels of *Wsi18, OsSMF1,* and *OsREM* from 300 K Rice Genome Microarray (www.ggbio.com). The transcript levels were measured in different sized panicles before heading (1, 3, 5, 8, 10, 15, 20, and 22 cm), at the indicated days after pollination (1, 3, 4, 11, and 21 days) and in the leaf, root, germinating seed, callus, and regenerating callus. (PPTX 70 kb)

Additional file 2: Figure S2. Rank analysis of OsSMF1 binding by Q9-PBM analysis. According to the rank-ordered signal distribution, two independent linear models (y = ax + b) were applied in the deep (b1 = 50320.3, slope = −38.3) and heavy right (b1 = 978.4, slope = −0.00657) tail regions of the curve. The extrapolated rank estimation for motif extraction was 1,286. (PPTX 84 kb)

Additional file 3: Table S2. Primers used for PCR/real-time PCR. (XLSX 12 kb)

Additional file 4: Figure S3. The query gene, *OsSMF1*, is marked by an asterisk. Each circle indicates a gene, and the lines represent the correlations between the genes. Eighty-five genes were identified as OsSMF1-related genes, with a minimum correlation value of 0.55 and depth of 1. (PPTX 473 kb)

Additional file 5: Figure S4. The expression patterns of transcription factors as a putative target of *OsSMF1* based on the 300 K Rice Genome Microarray (www.ggbio.com). The expression was measured in different sized panicles before heading (1, 3, 5, 8, 10, 15, 20, and 22 cm), at the indicated days after pollination (1, 3, 4, 11, and 21 days) and in the leaf, root, germinating seed, callus, and regenerating callus. (PPTX 75 kb)

Additional file 6: Table S1. List of putative OsSMF1 target genes, as determined by RAN analysis (r-value ≥ 0.55, depth = 1). (XLSX 19 kb)

## Abbreviations

OsSMF1: A basic leucine zipper transcription **f**actor involved in the regulation of rice **s**eed **m**aturation
PBM: Protein-binding microarrays
RAN: RiceArrayNet
SSP: Seed storage protein
