# Supplementary material for: Genome-wide identification of grain filling genes regulated by the OsSMF1 transcription factor in rice
Source: Rice (N Y). 2017 Apr 26;10:16. doi: 10.1186/s12284-017-0155-4 (PMC5405039; doi:10.1186/s12284-017-0155-4)
Supplement: Supplementary file 1 — Transcript levels of Wsi18, OsSMF1, and OsREM from 300 K Rice Genome Microarray (www.ggbio.com). The transcript levels were measured in different sized panicles before heading (1, 3, 5, 8, 10, 15, 20, and 22 cm), at the indicated days after pollination (1, 3, 4, 11, and 21 days) and in the leaf, root, germinating seed, callus, and regenerating callus. (PPTX 70 kb) [file 12284_2017_155_MOESM1_ESM.pptx]

## Slide 1
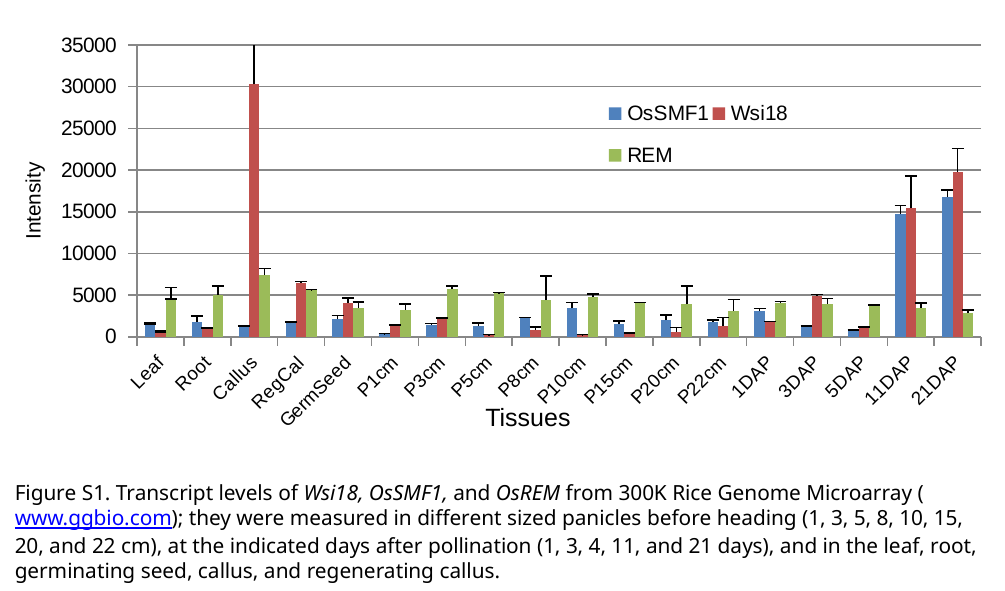

### Chart
| Category | OsSMF1 | Wsi18 | REM |
|---|---|---|---|
| Leaf | 1511.5 | 613.5 | 4541.0 |
| Root | 1801.0 | 1055.5 | 4994.5 |
| Callus | 1238.5 | 30284.5 | 7417.5 |
| RegCal | 1626.0 | 6460.5 | 5599.0 |
| GermSeed | 2121.0 | 4084.0 | 3406.5 |
| P1cm | 310.0 | 1369.0 | 3261.5 |
| P3cm | 1417.5 | 2230.0 | 5681.5 |
| P5cm | 1260.5 | 228.5 | 5222.0 |
| P8cm | 2247.0 | 868.5 | 4432.5 |
| P10cm | 3423.5 | 268.0 | 4836.5 |
| P15cm | 1555.5 | 290.0 | 4018.5 |
| P20cm | 2001.0 | 622.5 | 3912.0 |
| P22cm | 1745.5 | 1356.0 | 3103.5 |
| 1DAP | 3060.0 | 1825.5 | 4020.5 |
| 3DAP | 1236.0 | 4892.0 | 3960.5 |
| 5DAP | 767.0 | 1161.0 | 3821.0 |
| 11DAP | 14711.0 | 15419.5 | 3491.5 |
| 21DAP | 16820.5 | 19809.0 | 2888.5 |Intensity
Tissues
Figure S1. Transcript levels of Wsi18, OsSMF1, and OsREM from 300K Rice Genome Microarray (www.ggbio.com); they were measured in different sized panicles before heading (1, 3, 5, 8, 10, 15, 20, and 22 cm), at the indicated days after pollination (1, 3, 4, 11, and 21 days), and in the leaf, root, germinating seed, callus, and regenerating callus.
