# Supplementary material for: Genome-wide identification of grain filling genes regulated by the OsSMF1 transcription factor in rice
Source: Rice (N Y). 2017 Apr 26;10:16. doi: 10.1186/s12284-017-0155-4 (PMC5405039; doi:10.1186/s12284-017-0155-4)
Supplement: Supplementary file 2 — Rank analysis of OsSMF1 binding by Q9-PBM analysis. According to the rank-ordered signal distribution, two independent linear models (y = ax + b) were applied in the deep (b1 = 50320.3, slope = −38.3) and heavy right (b1 = 978.4, slope = −0.00657) tail regions of the curve. The extrapolated rank estimation for motif extraction was 1,286. (PPTX 84 kb) [file 12284_2017_155_MOESM2_ESM.pptx]

## Slide 1
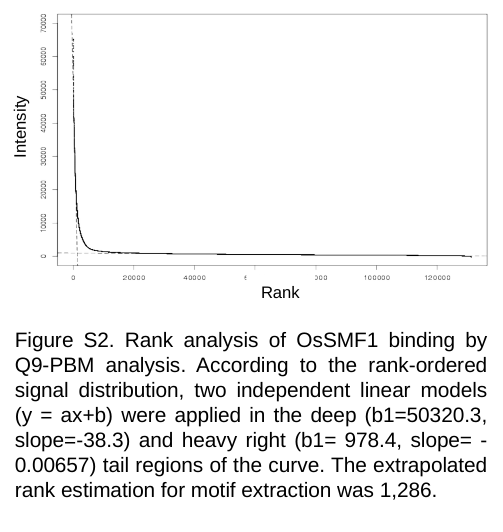

Intensity
Rank
Figure S2. Rank analysis of OsSMF1 binding by Q9-PBM analysis. According to the rank-ordered signal distribution, two independent linear models (y = ax+b) were applied in the deep (b1=50320.3, slope=-38.3) and heavy right (b1= 978.4, slope= -0.00657) tail regions of the curve. The extrapolated rank estimation for motif extraction was 1,286.
