# Supplementary material for: Genome-wide identification of grain filling genes regulated by the OsSMF1 transcription factor in rice
Source: Rice (N Y). 2017 Apr 26;10:16. doi: 10.1186/s12284-017-0155-4 (PMC5405039; doi:10.1186/s12284-017-0155-4)
Supplement: Supplementary file 4 — The query gene, OsSMF1, is marked by an asterisk. Each circle indicates a gene, and the lines represent the correlations between the genes. Eighty-five genes were identified as OsSMF1-related genes, with a minimum correlation value of 0.55 and depth of 1. (PPTX 473 kb) [file 12284_2017_155_MOESM4_ESM.pptx]

## Slide 1
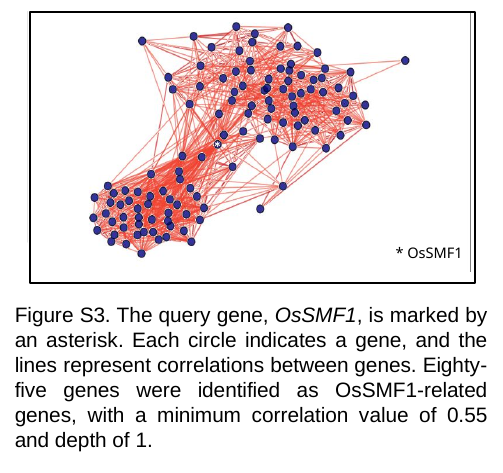

* OsSMF1
Figure S3. The query gene, OsSMF1, is marked by an asterisk. Each circle indicates a gene, and the lines represent correlations between genes. Eighty-five genes were identified as OsSMF1-related genes, with a minimum correlation value of 0.55 and depth of 1.
