# Supplementary material for: Genome-wide identification of grain filling genes regulated by the OsSMF1 transcription factor in rice
Source: Rice (N Y). 2017 Apr 26;10:16. doi: 10.1186/s12284-017-0155-4 (PMC5405039; doi:10.1186/s12284-017-0155-4)
Supplement: Supplementary file 5 — The expression patterns of transcription factors as a putative target of OsSMF1 based on the 300 K Rice Genome Microarray (www.ggbio.com). The expression was measured in different sized panicles before heading (1, 3, 5, 8, 10, 15, 20, and 22 cm), at the indicated days after pollination (1, 3, 4, 11, and 21 days) and in the leaf, root, germinating seed, callus, and regenerating callus. (PPTX 75 kb) [file 12284_2017_155_MOESM5_ESM.pptx]

## Slide 1
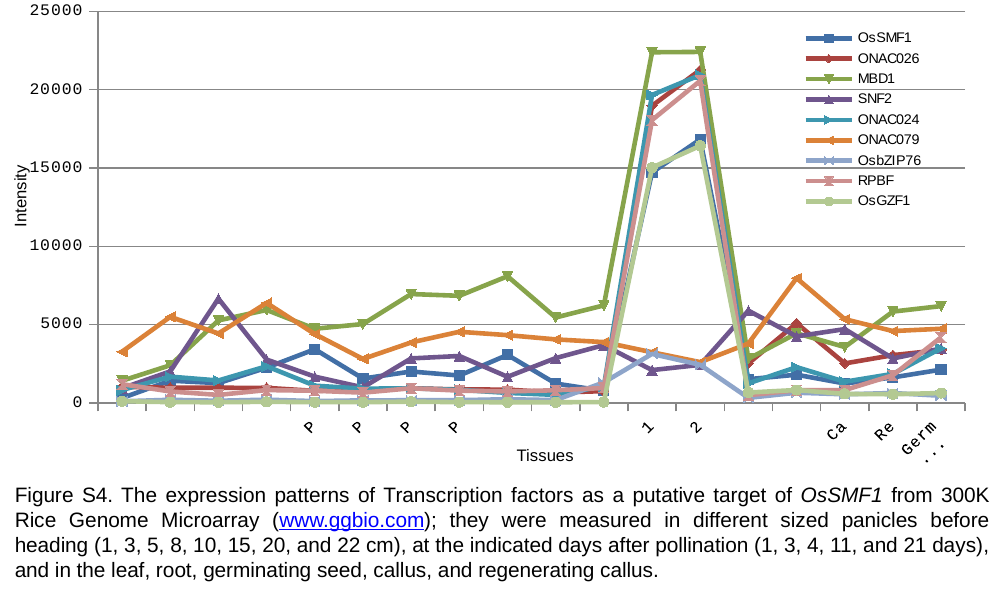

### Chart
| Category | OsSMF1 | ONAC026 | MBD1 | SNF2 | ONAC024 | ONAC079 | OsbZIP76 | RPBF | OsGZF1 |
|---|---|---|---|---|---|---|---|---|---|
| P1cm | 310.0 | 1024.0 | 1415.0 | 935.0 | 822.0 | 3266.0 | 104.0 | 1196.0 | 111.0 |
| P3cm | 1418.0 | 966.0 | 2403.0 | 2010.0 | 1673.0 | 5494.0 | 197.0 | 727.0 | 44.0 |
| P5cm | 1260.0 | 965.0 | 5266.0 | 6655.0 | 1429.0 | 4432.0 | 136.0 | 511.0 | 42.0 |
| P8cm | 2247.0 | 959.0 | 5931.0 | 2765.0 | 2336.0 | 6377.0 | 206.0 | 819.0 | 67.0 |
| P10cm | 3423.0 | 767.0 | 4731.0 | 1672.0 | 1095.0 | 4392.0 | 114.0 | 775.0 | 47.0 |
| P15cm | 1555.0 | 837.0 | 5033.0 | 988.0 | 908.0 | 2805.0 | 163.0 | 656.0 | 46.0 |
| P20cm | 2001.0 | 936.0 | 6946.0 | 2841.0 | 925.0 | 3858.0 | 166.0 | 936.0 | 79.0 |
| P22cm | 1746.0 | 858.0 | 6835.0 | 2992.0 | 832.0 | 4539.0 | 175.0 | 781.0 | 47.0 |
| 1DAP | 3060.0 | 850.0 | 8082.0 | 1665.0 | 635.0 | 4326.0 | 234.0 | 730.0 | 41.0 |
| 3DAP | 1236.0 | 659.0 | 5453.0 | 2858.0 | 508.0 | 4055.0 | 179.0 | 806.0 | 38.0 |
| 5DAP | 767.0 | 751.0 | 6225.0 | 3690.0 | 1056.0 | 3882.0 | 1310.0 | 951.0 | 51.0 |
| 11DAP | 14711.0 | 18965.0 | 22381.0 | 2095.0 | 19654.0 | 3231.0 | 3127.0 | 18052.0 | 15020.0 |
| 21DAP | 16821.0 | 21259.0 | 22402.0 | 2426.0 | 20933.0 | 2600.0 | 2426.0 | 20563.0 | 16410.0 |
| Leaf | 1512.0 | 2513.0 | 2808.0 | 5887.0 | 1245.0 | 3785.0 | 321.0 | 493.0 | 661.0 |
| Root | 1801.0 | 5082.0 | 4443.0 | 4245.0 | 2280.0 | 7960.0 | 652.0 | 804.0 | 822.0 |
| Callus | 1238.0 | 2518.0 | 3564.0 | 4712.0 | 1351.0 | 5341.0 | 546.0 | 816.0 | 572.0 |
| RegCal | 1626.0 | 3046.0 | 5830.0 | 2835.0 | 1849.0 | 4584.0 | 629.0 | 1763.0 | 548.0 |
| Germ
Seed | 2121.0 | 3380.0 | 6178.0 | 3475.0 | 3488.0 | 4731.0 | 452.0 | 4183.0 | 630.0 |Intensity
Tissues
Figure S4. The expression patterns of Transcription factors as a putative target of OsSMF1 from 300K Rice Genome Microarray (www.ggbio.com); they were measured in different sized panicles before heading (1, 3, 5, 8, 10, 15, 20, and 22 cm), at the indicated days after pollination (1, 3, 4, 11, and 21 days), and in the leaf, root, germinating seed, callus, and regenerating callus.
